# Engaging patients and parents to improve mental health intervention for youth with rheumatological disease

**DOI:** 10.1186/s12969-021-00503-7

**Published:** 2021-02-23

**Authors:** Oluwatunmise A. Fawole, Michelle V. Reed, Julia G. Harris, Aimee Hersh, Martha Rodriguez, Karen Onel, Erica Lawson, Tamar Rubinstein, Kaveh Ardalan, Esi Morgan, Anne Paul, Judy Barlin, R. Paola Daly, Mitali Dave, Shannon Malloy, Shari Hume, Suzanne Schrandt, Laura Marrow, Angela Chapson, Donna Napoli, Michael Napoli, Miranda Moyer, Vincent Delgaizo, Ashley Danguecan, Emily von Scheven, Andrea Knight, for the CARRA Investigators

**Affiliations:** 1 Children’s Hospital of Philadelphia, Philadelphia, PA USA; 2 University of Pennsylvania, Philadelphia, PA USA; 3 New York University Grossman School of Medicine, New York, NY USA; 4 University of Missouri-Kansas City, Children’s Mercy Kansas City, Kansas City, MO USA; 5 University of Utah, Salt Lake City, UT USA; 6 Riley Hospital for Children at Indiana University Health, Indianapolis, IN USA; 7 Hospital for Special Surgery, Weill Cornell Medicine, New York, NY USA; 8 University of California San Francisco, San Francisco, CA USA; 9 Albert Einstein College of Medicine, Children’s Hospital at Montefiore, Bronx, NY USA; 10 Ann & Robert H. Lurie Children’s Hospital of Chicago, Chicago, IL USA; 11 Northwestern University Feinberg School of Medicine, Chicago, IL USA; 12 Duke University Medical Center, Durham, NC USA; 13 Cincinnati Children’s Hospital Medical Center, University of Cincinnati, Cincinnati, OH USA; 14 Lupus Foundation of America, Washington, D.C USA; 15 Cure JM Foundation, Leesburg, VA USA; 16 Arthritis Foundation, Atlanta, GA USA; 17 Patient-Centered Outcomes Research Institute, Washington, D.C USA; 18 The Childhood Arthritis and Rheumatology Research Alliance, Milwaukee, WI USA; 19 Division of Rheumatology, Hospital for Sick Children, Toronto, ON M5G1X8 Canada; 20 University of Toronto Faculty of Medicine, Toronto, ON Canada

**Keywords:** Children, Mental health, Patient-engaged approach, Rheumatology, Mental health interventions, Survey

## Abstract

**Background:**

Mental health disorders are common in youth with rheumatological disease yet optimal intervention strategies are understudied in this population. We examined patient and parent perspectives on mental health intervention for youth with rheumatological disease.

**Methods:**

We conducted a mixed methods cross-sectional study, via anonymous online survey, developed by researchers together with patient/parent partners, to quantitatively and qualitatively examine youth experiences with mental health services and resources in North America. Patients ages 14–24 years with juvenile idiopathic arthritis, juvenile dermatomyositis, or systemic lupus erythematous, and parents of patients ages 8–24 with these diseases were eligible (not required to participate in pairs). Participants self-reported mental health problems (categorized into clinician-diagnosed disorders vs self-diagnosed symptoms) and treatments (e.g. therapy, medications) received for the youth. Multivariate linear regression models compared patient and parent mean Likert ratings for level of: i) comfort with mental health providers, and ii) barriers to seeking mental health services, adjusting for potential confounders (patient age, gender, disease duration, and patient/parent visual analog score for disease-related health). Participants indicated usefulness of mental health resources; text responses describing these experiences were analyzed by qualitative description.

**Results:**

Participants included 123 patients and 324 parents. Patients reported clinician-diagnosed anxiety (39%) and depression (35%); another 27 and 18% endorsed self-diagnosed symptoms of these disorders, respectively. 80% of patients with clinician-diagnosed disorders reported receiving treatment, while 11% of those with self-diagnosed symptoms reported any treatment. Patients were less comfortable than parents with all mental health providers. The top two barriers to treatment for patients and parents were concerns about mental health providers not understanding the rheumatological disease, and inadequate insurance coverage. Over 60% had used patient mental health resources, and over 60% of these participants found them to be helpful, although text responses identified a desire for resources tailored to patients with rheumatological disease.

**Conclusion:**

Self-reported mental health problems are prevalent for youth in this sample with rheumatological disease, and obstacles to mental health treatment include disease-related and logistic factors. Strategies are needed to improve acceptance and accessibility of mental health intervention, including routine mental health screening and availability of disease-specific mental health resources.

## Background

Patients with rheumatological diseases such as juvenile idiopathic arthritis (JIA), juvenile dermatomyositis (JDM), or childhood-onset systemic lupus erythematous (cSLE) may experience high psychological burden associated with their disease. Reported prevalence rates of depression and anxiety range from 15 to 65% [1]. Mental health disorders are associated with adverse outcomes in these patients, including decreased quality of life, suboptimal medication adherence, increased disease activity and pain, and challenges with transition to adult care [2–7]. Despite studies showing efficacy of mental health interventions in pediatric rheumatology patients [8–10], it has been recognized that mental health problems are often underdiagnosed and untreated. According to a 2015 survey of pediatric rheumatologists in the Childhood Arthritis & Rheumatology Research Alliance (CARRA), a collaborative research alliance which includes 90% of pediatric rheumatologists in North America, 52% of respondents reported inadequate mental health symptom identification and 45% reported inadequate treatment of depression and anxiety amongst their patients with cSLE [11]. These findings were echoed by a mixed methods study of behavioral health providers, who identified several gaps in mental health care for pediatric rheumatology patients, including lack of protocols for screening, intervention, and follow-up, a need for integration of mental health providers into medical care, and better access to peer support resources for patients and families [12].

Understanding patient and parent perspectives on mental health care is essential to assessing patients’ health status and service gaps [13–16]. To our knowledge, there has only been one qualitative single center study looking at patient and parent perspectives on barriers and facilitators to addressing mental health needs in youth with rheumatological disease; it found that pediatric rheumatologists were perceived as a preferred source for mental health care referral and resources [17]. Additionally, there is emerging recognition of the importance of patient and parent engagement in pediatric chronic disease research in order to maximize the relevance, quality, and acceptability of study findings [18, 19]. Involving patients and families as members of the research team in the design, implementation and interpretation of studies (i.e. using a patient-engaged approach) is a valuable strategy for exploring sensitive topics such as mental health.

The objective of this study was to investigate patient and parent perspectives on mental health intervention for youth with rheumatological disease, using a patient-engaged approach. Specifically, we conducted a mixed methods study to: i) characterize experiences and perceptions about mental health services and resources for patients with JIA, JDM or cSLE, ii) identify perceived barriers to utilization of these services, and iii) compare patient and parent perspectives for these responses.

## Methods

### Study design

We conducted an anonymous, online cross-sectional survey of patients with rheumatological disease, and parents. A patient-engaged approach was used from development through conduction of the study, leveraging the Patients, Advocates, and Rheumatology Teams Network for Research and Service (PARTNERS), a patient-powered research network funded by the Patient-Centered Outcomes Research Institute (PCORI), which includes the CARRA network, the Pediatric Rheumatology Care and Outcomes and Improvement Network (PR-COIN), and 3 patient organizations (Arthritis Foundation, Lupus Foundation of America, and Cure JM Foundation). The PARTNERS network formally links pediatric rheumatologists, researchers, and JIA, JDM, and cSLE patients and family members, who are central collaborators in the research. The survey was drafted by the CARRA SLE Mental Health Workgroup in collaboration with three patient and three parent advisors, who contributed critical feedback surrounding inclusion of response item options to capture the range of mental health experiences, in addition to content guided by the literature [7, 11, 17, 20]. The survey was then iteratively refined to incorporate input from these advisors, and representatives from the other organizations in PARTNERS. The study was approved by the Institutional Review Boards for the Children’s Hospital of Philadelphia (CHOP), Duke University, the Lupus Foundation of America, and the Hospital for Sick Children.

### Participants & setting

Eligible patients were ages 14–24 years reporting a diagnosis of JIA, JDM or cSLE, and receipt of specific treatment [including: non-steroidal anti-inflammatory drugs (NSAIDs); glucocorticoids (oral, intravenous, or intra-articular); conventional disease modifying anti-rheumatic drugs (DMARDs); and biologic DMARDs]. Parents of patients ages 8–24 years were also eligible to participate; to facilitate anonymity, they were not required to participate in pairs with their children. Age inclusion criteria were generally aligned with the definition of youth according to the US Department of Health and Human Services Healthy People 2020 Objectives (which includes adolescents ages 10–17 and young adults ages 18–25) [21]. However, we utilized the following modifications: lower age of 14 years for patients to enable consent for their own participation, and facilitate anonymous response; eligibility of parents of patients ages 8 and older, based on parent advisor feedback regarding the importance of characterizing mental health for pre-adolescent patients.

The survey was conducted anonymously to maximally elicit personal information about mental health, while reducing underreporting or misreporting which can occur due to social desirability bias around sensitive topics [22]. To recruit participants living in the United States and Canada, we leveraged the patient organizations in the PARTNERS network (Arthritis Foundation, Lupus Foundation of America, and Cure JM Foundation). Participants accessed the REDCap survey via weblink between August 2017 to August 2018, sent via email listservs, and available on websites and social media sites for the above 3 patient organizations; the weblink was also available via local advertisements at nine participating CARRA clinics. Data was managed using the secure, REDCap web application designed to support data capture for research [23].

### Survey instrument

The anonymous online survey, available in English and Spanish (using forward and back-translation methods), comprised quantitative and qualitative items to examine experiences with mental health care for patients with JIA, JDM or cSLE. The survey captured the following patient disease characteristics: rheumatological diagnosis, medications, disease duration and activity (active vs inactive), visual analog score (VAS) for disease-related health (0 = very well and 10 = very poor). Active disease was defined across disease groups by an affirmative response to both of the following the questions: “Is your disease currently active (not under good control, with or without medication)?”, and “Has your rheumatologist told you that your disease is currently active/ not active?”. Patient demographics included age, gender, race, ethnicity, household income and education level, insurance, and location [United States census region used by the Centers for Disease Control and Prevention [24], or Canada (regions not included due to small Canadian sample]. Participants also provided mental health history including: clinician-diagnosed mental health disorders (by a rheumatologist, psychiatrist, pediatrician/family care doctor, other doctor, or psychologist), past or current self-diagnosed mental health symptoms, and past or current mental health treatment received (e.g. therapy or counseling, psychotropic medications, or inpatient psychiatric care). Additionally, in response to a hypothetical vignette simulating a situation of emotional distress for the patients (Additional File 1), participants used a Likert scale to rate their: i) level of comfort with potential mental health providers (0 = very uncomfortable, 4 = very comfortable), and ii) level of concern about potential barriers (16 for patients, 17 for parents) to seeking help from a mental health professional (0 = not at all, 4 = extremely), based on known correlates of unmet need for mental health services in children and adolescents [20, 25]. We also collected information on the broad scope of types of mental health resources that participants had used (including general counseling services, peer support groups, camps, online resources, and printed handouts/resources). We asked participants who had used these resources if they found them to be helpful, not helpful, or if they were unsure if the resource was helpful. These participants were then asked to provide a qualitative text entry explaining their response.

### Statistical analysis

Participant characteristics were summarized using frequencies and proportions for categorical variables, and means and standard deviations or medians and interquartile ranges for continuous variables, as appropriate. Ineligible responders and incomplete responses (from unsubmitted surveys) were excluded. We measured the prevalence of self-reported mental health problems for patients with rheumatological disease, stratifying responses by mutually exclusive groups for clinician-diagnosed disorders versus self-diagnosed symptoms, and tabulated reported mental health treatments received by these groups. For participants indicating use and helpfulness of mental resources, qualitative text responses were analyzed using qualitative description to summarize positive or negative experiences with mental health resources, and illustrative text responses were selected. Qualitative description is a low-inference approach that presents the facts from exactly the informants’ point of view. It is the qualitative method of choice when only a description of a phenomenon is desired, and is particularly useful in health care research to focus on the patient experience, for the purpose of needs assessment and intervention development [26–28].

We calculated mean Likert scores for level of comfort with potential mental health providers, and frequency of barriers to seeking mental health treatment. Using multivariate linear regression to control for potential confounders (patient age, gender, disease duration, and patient/parent VAS), we used two separate models to compare patient and parent responses for ratings for comfort level with mental health providers, and frequency of barriers to mental health treatment. We also conducted two stratified regression analyses to compare responses by:1) patient age group (14–17 versus 18–24 years) to determine if the results differed between adolescents and young adults; and 2) presence or absence of reported mental health problem (either clinician- or self-diagnosed), to determine if actual experience with a mental problem impacted the results. Analysis was conducted using Stata^(R)^ Data Analysis and Statistical Software version 15.0. Alpha ≤0.05 denoted a statistically significant result.

## Results

The survey was initiated by 749 respondents. The following respondents were excluded in a sequential fashion: 53 who did not indicate consent; 63 who did not indicate an eligible rheumatological diagnosis; 44 who did not indicate specific treatment; 10 indicating age above the eligible range; 132 with incomplete mental health survey items. This resulted in a final survey sample of 447.

### Subject characteristics

Four hundred forty-seven respondents included 123 patients and 324 parents. Nine respondents (2%) completed the Spanish version of the survey. The most prevalent diagnosis was JIA for both patient (41%) and parent (49%) respondents, followed by JDM (35 and 40%), and cSLE (24 and 11%). The most common treatments received for rheumatological disease were conventional disease-modifying anti-rheumatic drugs (DMARDs) by > 90% of patients. For JIA participants, ever use of methotrexate (80%) and oral glucocorticoids (54%) approximated that for large CARRA JIA Registry cohort (73 and 47%) [29]. Additional disease and demographic characteristics are shown in Table 1.

**Table 1:** Characteristics of patients with rheumatologic disease reported by patients and parents

Patient Characteristic	Patient Report, *N* = 123	Parent Report, *N* = 324
Rheumatologic diagnosis		
Juvenile idiopathic arthritis (JIA)	50 (41)	160 (49)
Juvenile dermatomyositis (JDM)	43 (35)	130 (40)
Systemic lupus erythematosus (cSLE)	30 (24)	34 (11)
Immunosuppressive medications ever taken^a^		
Conventional disease-modifying anti-rheumatic drugs (DMARDS)	114 (93)	293 (90)
Biologic DMARDS	65 (53)	143 (44)
Glucocorticoids (oral, intravenous or intra-articular)	113 (92)	256 (79)
Active disease status (Y/N)	68 (55)	167 (52)
Visual analog score of disease-related health	3.6 (1.8, 6.2)	2.9 (1.3, 5.1)
Disease duration, years	8 (5, 12)	5 (2, 8)
Age, years	19 (16, 23)	13 (10, 17)
Age group		
8 to 11	0 (0)	112 (35)
12 to 17	46 (37)	145 (45)
18 to 24	77 (63)	67 (21)
Female	115 (94)	235 (73)
Race		
Asian	8 (7)	10 (3)
Black or African American	3 (2)	15 (5)
White	96 (78)	264 (81)
Other	16 (13)	35 (11)
Hispanic/Latino ethnicity	15 (13)	37 (12)
Household income above federal poverty line	99 (80)	288 (89)
Highest household education level		
Below College	35 (28)	62 (19)
College Degree	56 (46)	136 (42)
Advanced Degree	30 (24)	126 (39)
Primary insurance for patient		
Public	19 (15)	49 (15)
Private	76 (62)	252 (78)
Other	8 (7)	17 (5)
None	1 (< 1)	6 (2)
Location		
Northeast US	34 (28)	56 (18)
Midwest US	22 (18)	77 (25)
West US	34 (28)	94 (27)
South US	25 (20)	65 (21)
Canada	1 (< 1)	7 (2)

Categorical variables are shown as number (percentage);continuous variables reported with median (interquartile range). There were significant differences in patient and parent responses for patient age (*p* < 0.0001), disease duration (*p* < 0.0001), and VAS (*p* = 0.01) by Wilcoxon rank-sum test, and for gender (*p* < 0.001) by Pearson χ^2^. The following variables had missing data: active disease status (*n* = 35), ethnicity (*n* = 9), poverty (*n* = 2), region (*n* = 32). Gender was reported to be “male” by *n* = 96 and “other” by *n* = 1

^a^Conventional DMARDs reported include: methotrexate (66% of patients and 79% of parents), hydroxychloroquine (58 and 39%) intravenous immunoglobulin (24 and 32%), mycophenolate (28 and 25%), less commonly azathioprine, cyclosporine, tacrolimus, cyclophosphamide, sulfasalazine and leflunomide, dapsone and colchicine. Biologic DMARDS include: etanercept (28 and 26%), adalimumab (18 and 18%), rituximab (19 and 9%), abatacept (8 and 8%), less commonly infliximab, tocilizumab, canakinumab, ustekinumab, anakinra, tofacitinib, certolizumab, golimumab, belimumab, secukinumab, and rilonacept. Glucocorticoid treatment reported includes oral/intravenous medication (89 and 74%) and joint injections (25 and 21%)

### Prevalence of reported mental health problems and treatments received

**Fig. 1:**
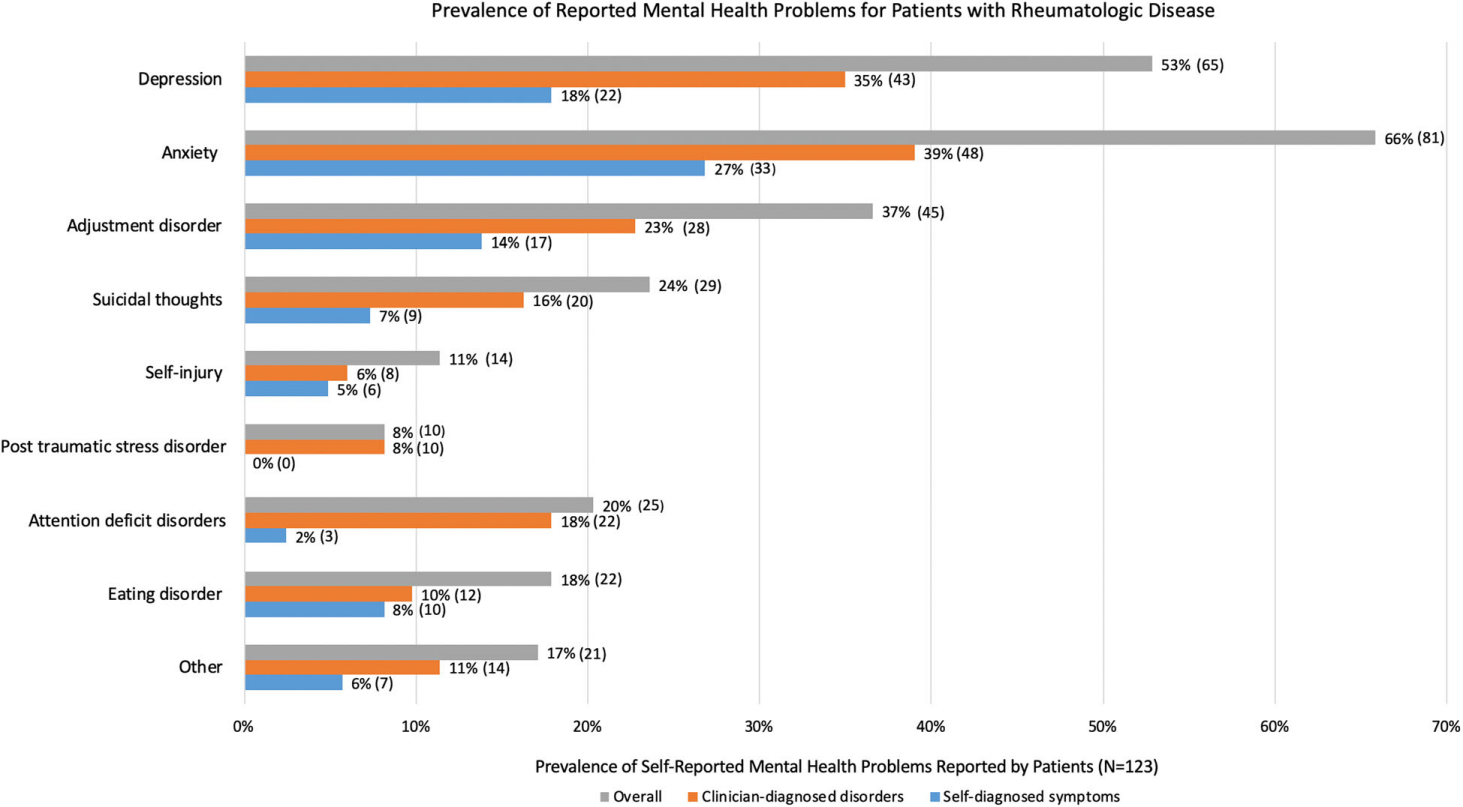
Results are shown for the prevalence of mental health problems reported by patients with rheumatological disease, categorized by clinician-diagnosed disorders and self-diagnosed symptoms. The category for other mental health problem includes bipolar disorder/psychosis, oppositional defiant disorder and substance abuse

A history of any clinician-diagnosed or self-diagnosed mental health problem among patients was reported by 92 patients (75%) and 201 parents (62%). Patients most commonly reported problems with anxiety (66%), depression (53%), and adjustment (37%) (Fig. 1), which paralleled those reported by parents (Table 2). History of suicidal thoughts (23%), attention deficit disorders (20%), and eating disorders (18%) were also frequently reported by patients. Adjusted models controlling for patient age, gender, disease duration, and patient/parent VAS showed no significant difference between patients and parents for reported mental health problems.

**Table 2:** Prevalence of Reported Mental Health Problems and Treatment Received Among Patients with Rheumatologic Disease

Prevalence of Reported Mental Health Problems Among Patients	Patient Report, *N* = 123	Parent Report, *N* = 324
	N(%)	N(%)
Any mental health problem	92 (75)	201 (62)
Depression	65 (53)	103 (32)
Anxiety	81 (66)	172 (53)
Adjustment disorder	46 (37)	95 (30)
Suicidal thoughts	28 (23)	40 (12)
Self-injury	15 (12)	18 (6)
Post-traumatic stress disorder	10 (8)	34 (10)
Bipolar disorder/ psychosis	12 (10)	7 (2)
Eating disorder	24 (20)	18 (6)3
Attention deficit disorder	25 (20)	66 (20)
Oppositional defiant disorder	2 (2)	18 (6)
Substance abuse/addiction to alcohol or drugs	5 (4)	4 (1)
Treatment Received by Patients with Mental Health Problems	Patient Report, *N* = 92	Patient Report, *N* = 201
Any mental health treatment	44 (48)	128 (65)
Psychotherapy/counseling	38 (41)	112 (57)
Location of services received		
Therapist office in community	25 (66)	87 (78)
School	7 (8)	14 (13)
Clinic at rheumatologist hospital/location	6 (16)	30 (27)
Psychiatric hospital/facility	4 (4)	3 (3)
Psychotropic medications^a^	30 (33)	64 (32)
Anti-depressants	23 (77)	41 (64)
Anti-anxiety	11 (37)	17 (27)
Other	2 (7)	19 (30)
I don’t know	4 (13)	2 (3)
Prescriber for those receiving medications^a^		
Primary care/family doctor	16 (53)	27 (42)
Community mental health provider	12 (40)	24 (38)
Mental health provider at rheumatologist hospital/location	2 (7)	11 (17)
Other specialist doctor	6 (20)	11 (17)
Inpatient Psychiatric Care	5 (5)	13 (7)

^a^The sum of percentages might exceed 100%, as the respondents were allowed to select all that applied

Of the 92 patients reporting a history of at least one mental health problem, 32 (35%) indicated a clinician-diagnosed disorder only, 37 (40%) indicated self-diagnosed symptoms only, and 17 (18%) indicated both a clinician-diagnosed disorder and self-diagnosed symptoms (6 did not specify). For every type of mental health problem, more than a third of patients reporting the problem had self-diagnosed symptoms rather than a clinician diagnosis, with the exception of post-traumatic stress disorder and attention deficit disorder (Fig. 1). For patients with mental health problems, 46% of patients indicated that their rheumatologist was aware of the problem, compared to 70% of parents (*p* < 0.0001, *Z* = -3.87, test of proportions).

Of patients with a reported mental health problem (including clinician and self-diagnosed), receipt of mental health treatment was reported by 44 of 92 (48%) patients and 128 of 201 (65%) parents (Table 2). Psychotherapy or counseling was the most common reported treatment modality, reported by 41% of patients and 57% of parents. About a third of patients reported receiving psychotropic medications, and < 10% of reported receiving inpatient psychiatric care. Of those reporting a clinician-diagnosed disorder, 39 of 49 (80%) patients reported receiving mental health treatment; parents indicated that 90 of 115 (78%) patients had received treatment. Of the 37 patients reporting self-diagnosed symptoms only, 4 (11%) reported receiving mental health treatment; parent report indicated that 27 of 62 (44%) received treatment.

### Attitudes & barriers for seeking mental health services

**Fig. 2:**
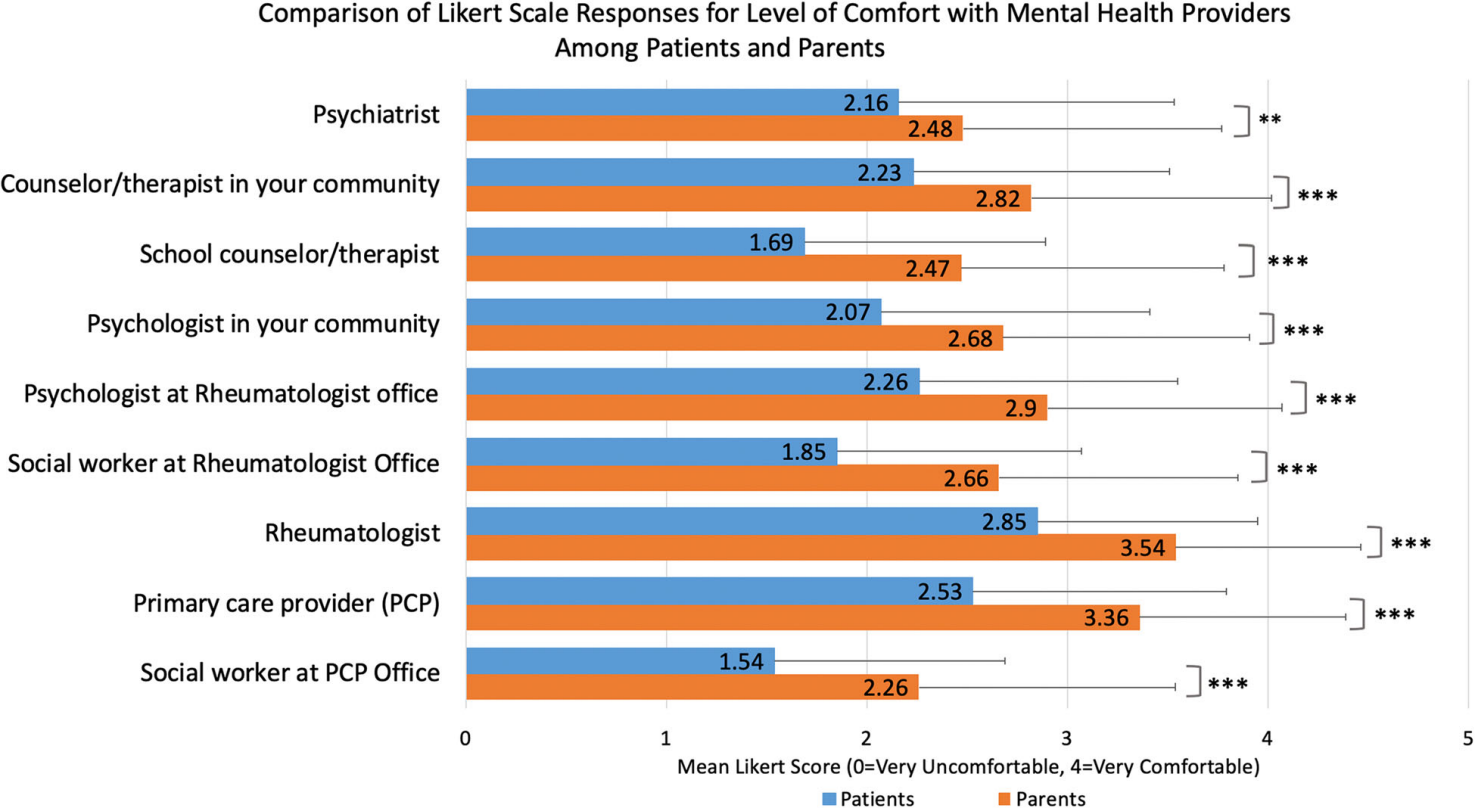
Results are shown for linear regression models comparing Likert ratings for level of comfort with mental health providers between patients and parents. Models were adjusted for patient age, gender, disease duration, and patient/parent visual assessment score for disease-related health. *p < 0.01, **p < 0.001, ***p < 0.0001. Beta coefficients were as follows for: psychiatrist (0.44), counselor/therapist in your community (0.65), school counselor/therapist (0.74), psychologist in your community (0.78), psychologist at rheumatologist office (0.60), social worker at rheumatologist office (0.85), rheumatologist (0.74), PCP (0.78), social worker at PCP office (0.81)

In response to the hypothetical vignette simulating a situation of emotional distress for youth with rheumatological disease, patients were significantly less comfortable seeking help from all potential mental health providers than parents, adjusting for patient age, gender, disease duration, and patient/parent VAS (Fig. 2). While both groups felt most comfortable with rheumatologists (mean Likert score for patients 2.85 vs parents 3.54, *p* < 0.0001) and primary care providers (PCPs) (patients 2.53 vs parents 3.36, *p* < 0.0001), they were least comfortable with social workers at the primary care (patients 1.54 vs parents 2.26, *p* < 0.0001) and rheumatologist office (patients 1.85 vs parents 2.66, *p* < 0.0001), and school counselors/therapists (patients 1.69 vs parents 2.47, *p* < 0.0001). There were no significant differences in level of comfort with mental health providers when respondents were stratified by patient age (14–17 vs 18–24). When stratified by reported mental health problem in the patients, those reporting a problem were significantly more comfortable interacting with counselors/therapists in the community than those without (2.72 vs 2.51, *p* = 0.038), adjusting for other potential confounders.

**Fig. 3:**
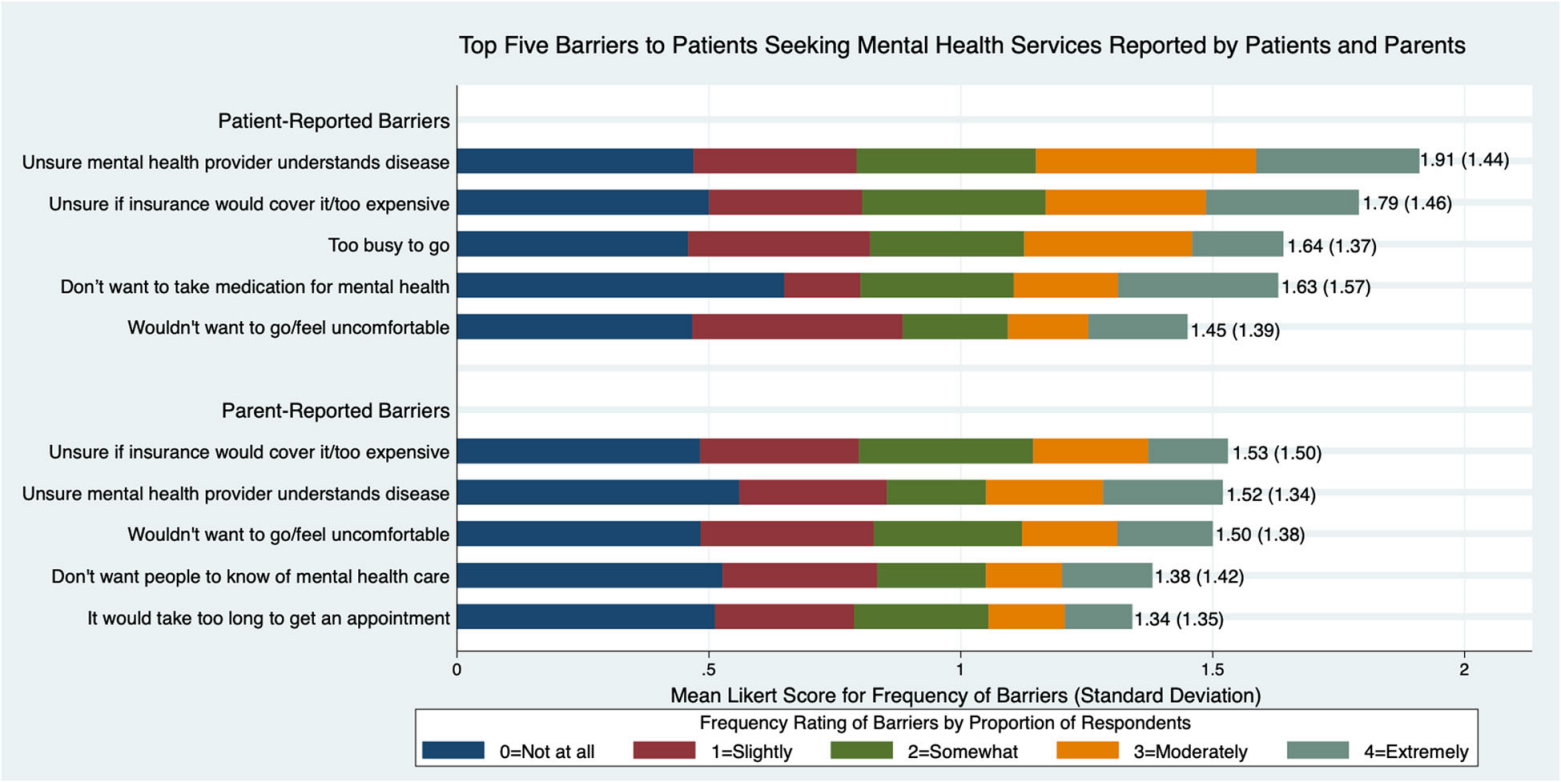
Results are shown for the top five barriers to seeking mental health services for patients, rated by patients and parents

In response to items for the hypothetical vignette regarding barriers to accessing mental health services (16 for patients, 17 for parents), patients and parents both rated the following in the top two barriers: concerns that mental health professionals would not understand the rheumatological disease; and that insurance coverage would be inadequate (Fig. 3). Another barrier rated among the top five by both patients and parents included patients not wanting to go/feeling uncomfortable with accessing mental health services. However, there were also statistically significant differences between patient and parent Likert ratings among the top five barriers, after adjusting for potential confounders. Specifically, patients reported more concern about not wanting to take medication for mental health problems (patient 1.63 vs parent 1.27, *p* = 0.01), whereas parents were more concerned that patients would not want others to find out about mental health treatment (patient 1.14 vs parents 1.38, *p* = 0.04). There were no significant differences in ratings when stratified by age group or presence of a mental health problem.

### Experiences with mental health resources

69% of patients (*n* = 85) and 63% of parents (*n* = 207) reported use of a patient mental health resource (Table 3), of which online informational resources were most common by patient report, and patient counseling services by parent report. Fifty-two (61%) patients and 148 (72%) parents thought the mental health resources were helpful.

**Table 3:** Reported Use of Mental Health Resources for Patients with Rheumatological Disease

Types of Mental Health Resources Used	Patient Report, *N* = 85	Parent Report, *N* = 207
	N(%)	N(%)
Online information	51 (60)	48 (23)
Counselor/therapist	38 (45)	105 (51)
Mental health brochures/books	27 (32)	56 (27)
Peer support groups	27 (32)	43 (21)
Psychologist	27 (32)	65 (31)
School guidance counselor	23 (27)	79 (38)
Camps with patients with a similar rheumatological condition	21 (25)	75 (36)
Psychiatrist	20 (24)	40 (19)
Social worker	19 (22)	36 (17)
Religious youth groups	15 (18)	42 (20)
Telephone-based mental health services	5 (6)	7 (3)
Other^a^	10 (12)	20 (10)

^a^Other includes family, close friends, including family and close friends in the mental health profession, academic conferences for pediatric rheumatological disease, art therapy, life coaches, and other specialist doctors

Illustrative quotes for qualitative text responses of experiences with patient mental health resources are shown in Table 4. Respondents described positive experiences with counselors who helped patients process emotions and pain, but several indicated negative experiences with counselors who did not understand the impact of the rheumatological disease on mental health. Other comments shed light on the high cost and scheduling/logistic challenges associated with counseling services. Respondents indicated that disease-specific online resources were informative, and those with social media communities were helpful; however, patients sometimes felt uncomfortable sharing very personal information through online social platforms, and found it challenging to be reminded of their struggles with rheumatological disease. Peer support groups were also identified as opportunities to hear from other patients experiencing similar problems, although some commented that it was sometimes difficult to overcome unease about sharing personal details about their lives. Summer camps were seen as beneficial because patients could be around people like them who understood what it was like to have a rheumatological disease. However, some patients reported not being able to engage in summer camps if their disease was too severe or if the camp did not have enough focus on their specific rheumatological disease (Table 4). Print books were also helpful, but noted to be scarce by some respondents.

**Table 4:** Illustrative Quotes for Experiences with Mental Health Resources for Patients with Rheumatologic Disease

Mental Health Resources Used	Helpful	Not Helpful/Unsure
Counseling	“The counselor helped me understand that what I was feeling was valid, and helped me put a name to the feelings and be able to process them.” –Patient, age 23 “The therapist helped him deal with school issues bothering him, how to handle his moods, how to optimize his time when not feeling well and helped us as a family have productive and more meaningful conversations.” –Parent for patient, age 24 “Our daughter likes having someone else to talk to other then her mom and dad. The therapist helped her cope with the injection and IV.” –Parent for patient, age 9 “Having a therapist to talk to about her pain and how it affects her has been very helpful.” –Parent for patient, age 8	“Therapy was extremely expensive and the therapist we saw was convinced our daughter had a specific genetic mutation never addressed her anger and mood swings.” –Parent for patient, age 8 “Counseling was very infrequent due to distance to connect with a psychologist who was knowledgeable on JDM.” –Parent for patient, age 9 “Child was not very compliant; Child did not ‘buy in to’ the therapy - said what he thought the therapist wanted to hear; didn’t absorb the tools he was learning to cope with anxiety/social anxiety. Therapist meant well, but didn’t completely ‘get’ JDM.” –Parent for patient, age 16 “I don’t like talking to strangers about specific things (like counseling or therapy) so I didn’t feel like it was helpful to me specifically.” –Patient, age 21
Peer Support Groups	“Support group made me think about worse cases related to Lupus, but also and most importantly my symptoms were understood.” –Patient, age 19 “It is nice to hear about other people going through similar and different situations. It is very lonely sometimes when no one around you really understands what is going on...it is only made worse when you don’t understand what is going on a lot of the times.” –Patient, age 24	“She attends youth group on a regular basis, thought I am not certain that she applies any content directly to her JIA diagnosis.” – Parent for patient, age 15 “It was called a lunch bunch group in elementary school for a group (about 5) children. I don’t believe any of the children opened up to others due to uneasiness/embarrassment. Did not last long-about 4 sessions.” –Parent for patient, age 13
Disease-related Camps	“Summer camp is amazing!! We talked about things that other people just don’t get.” –Patient, age 14 “Camp was amazing. Being around ‘people like me’ is one of the things she loved about it. If she needed to stop an activity and wrap her ankle no one made a big deal about it.” –Parent for patient, age 15 “Camps/conferences where families attend have been extremely helpful.” –Parent for patient, age 13	“The camp I attended was for kids with JA and JDM, and because my disease has been so severe, I did not relate or grow close to them.” –Patient, age 18 “The camp was for people with all types of rheumatic diseases, so I was not able to get close to them.” –Patient, age 18
Online Resources	“I recently found a group of kids whom also have JDM and have a private page set up on Facebook. It was really nice when I found it because it showed me I wasn’t alone and other people are facing similar issues.” —Patient, age 14 “The Cure JM website and associated Juvenile Myositis Australia Inc. have been incredibly supportive and informative.” –Parent for patient, age 8	**“**The online support group is nice for talking, but I don’t feel comfortable saying all that is going on.” –Patient, age 18 “In the [online] group people support each other and give advice to one another. However, I do not visit the group page that often because being reminded that I have JDM and the struggles of being sick would dampen my mood.” —Patient, age 14
Print Handouts/Resources	“The ‘Myositis and You’ book was incredibly helpful and still is to this day. A must have for everyone newly diagnosed.” –Parent for patient, age 8	“Those resources, besides online, are not available in our area/smaller areas.” –Parent for patient, age 17

## Discussion

To our knowledge, this is the largest study focusing on mental health needs for youth with rheumatological disease from the perspective of patients and parents, yielding several key findings to guide improvement of mental health intervention. First, our sample reported a high prevalence of mental health problems affecting the patients, including both clinician-diagnosed disorders and a notable proportion of self-diagnosed symptoms. While most patients with a clinician-diagnosed disorder had received mental health services, more than half with self-diagnosed mental health symptoms had not. Second, patients were less comfortable than parents with all mental health providers, and there was a greater discomfort with typical mental health providers (such as counselors/therapists, social workers, psychologists) for both groups. Additionally, key barriers to seeking mental health treatment for both patients and parents included concern about lack of disease understanding by mental health providers, and lack of adequate insurance coverage due to high cost of mental health services. Finally, although participants reported use of several helpful mental health resources, they desired more disease-specific resources to provide specialized and individualized support. These important insights provide several targets for improving mental health education and resource utilization, through collaborative relationships between youth with rheumatological disease, their families, medical and mental health providers. These targets are also aligned with those of the recent American Board of Pediatrics Roadmap Project (https://www.abp.org/foundation/roadmap), which acknowledges the substantial mental health needs of patients and families with chronic pediatric health conditions, and endeavors to address these needs through development of mental health tools and resources.

Anxiety and depression were the most prevalent clinician-diagnosed mental health disorders amongst survey respondents at 39 and 35%, respectively, which are also the most prevalent mental health disorders in the general youth population at 8 and 20% [30, 31]. Our findings are supported by other studies of youth with rheumatological disease [4, 5, 7], and parallel reported depression and anxiety rates for other pediatric chronic disease populations [32, 33]. We also expand understanding of the mental health burden for youth with rheumatological disease, as adjustment disorder, attention-deficit disorder, suicidal thoughts, and eating disorder were commonly reported. The high rate of reported mental health problems for youth with rheumatological disease underscores the need for routine mental health screening in this population, particularly because patients might not feel comfortable reporting their mental health symptoms to their parents or other care providers.

While it is encouraging that most (80%) of the patients with clinician-diagnosed mental health disorders were reported to have received treatment, the proportion was much lower for substantial group with self-diagnosed symptoms only, and most pronounced for patient vs parent report (11% vs 44%). Providing mental health education to youth with rheumatological disease may help increase perceived usefulness and benefits of mental health services. Our participants found online and print resources to be helpful, and educational resources are known to be a facilitator for youth seeking out mental health services [34, 35]. Enhancing the ability of medical providers to communicate with patients about mental health may also promote acceptability and reduce stigma amongst patients who may be considering mental health care. Both patients and parents reported the highest level of comfort with rheumatologists and PCPs, likely due to the high level of trust and familiarity that these clinicians have with the rheumatological diagnosis [17]. Yet, many of these medical providers may not be trained in mental health assessment and intervention; this represents a gap in care, for which efforts to provide primary care and pediatric subspecialty physician training have been initiated by the American Board of Pediatrics Roadmap Project [36]. Additionally, as the majority of patients who received treatment had accessed psychotherapy/counseling and psychotropic medication through PCP and community services, our results highlight the importance of communication and partnership between PCPs and rheumatologists to facilitate mental health intervention for patients.

Patient and parent discomfort with social workers, psychologists, and counselors/therapists might be due to stigma surrounding receiving care from mental health providers [37]. Stigma and label avoidance (unwillingness of those suffering from mental health conditions to label them as such) are well-described barriers to those with (or at risk for) mental health concerns in terms of accessing treatment options such as counseling and/or medication [38–41]. Additionally, specific to the pediatric rheumatology context, a prior study found that patients have limited or no access to mental health providers in North American pediatric rheumatology clinics, and that these services are not typically presented as a standard part of comprehensive rheumatological care [12]. This is supported by our findings that of those who used mental health resources for patients, only 22% of patients and 17% of parents had accessed a social worker. Although social workers may often be the most accessible of these providers, patients and families may be unaware of the role that they can play in facilitating and providing mental health resources, as many social workers believe that they are underutilized in this capacity [12]. Increased availability of social workers, psychologists and counselors in the pediatric rheumatology setting, as well as integration and normalization of their services as a part of standard rheumatological care may increase both access and acceptance of mental health treatment. For example, in the United Kingdom, the national Standards of Care for Pediatric Rheumatology include recommendation for clinical psychologists to be core members of the multidisciplinary team, and the National Health Service of England requires formally commissioned tertiary level pediatric rheumatology care centers to have an embedded mental health professional [42, 43]. These policies should be considered within the North America and other regions, as they are likely to increase familiarity of patients and parents with mental health providers, and improve trust and utilization of these mental health providers.

It is noteworthy that patients and parents were concordant in identifying the top two barriers to seeking mental health services; these were concerns about a lack of understanding by mental health providers about the rheumatological disease, and about lack of adequate insurance coverage. While the latter finding is a known general barrier from existing literature [37, 44], the concern about lack of understanding of disease is particularly relevant to providing effective intervention to youth with rheumatological disease. These results suggest that even for those who can overcome insurance issues to access mental health services, there remains a need to address disease-specific issues within the course of mental health treatment. This finding was echoed in the text responses regarding mental health resources, with several respondents reflecting on the reduced helpfulness of such resources due to a lack of disease-specific content. These results indicate a clear need for affordable mental health care options that are also disease-informed, to address the nuanced mental health challenges faced by patients with rheumatological conditions. Interventions to address this need may include co-location of mental health providers in pediatric rheumatology settings, educational initiatives to improve community mental health provider knowledge of pediatric rheumatological conditions, and development of disease-specific peer support and informational mental health resources. Encouragingly, mental health resources for pediatric chronic disease patients and families are available from the American Board of Pediatrics Roadmap Project, and more are being developed, with acknowledgement that they may benefit from tailoring to specific diseases and individual patient needs.

### Strengths & Limitations

The strengths of this study include the engagement of patients and parents in the survey design, which was critical to constructing survey items with language conducive to inquiring about the sensitive topic of mental health. Engagement of a patient advocacy group such as PARTNERS was also essential to optimizing the content, design, advertisement and distribution of the survey to reach a broad, national audience of patients and parents through social media and other approaches. Administration of a survey in both English and Spanish also aimed to increase representativeness.

This study also has limitations. First, since the survey responses were self-reported and anonymous, there is possibility of misclassification. We were not able to definitively confirm clinician diagnoses of rheumatological disease; however, we think that our sample represents the intended patient population, as reported medications were similar to expected, with treatment use in JIA participants approximating that in the large CARRA JIA Registry cohort [29]. We were also not able to confirm reported mental health diagnoses, nor ask questions surrounding severity of reported mental health problems, due to lack of a mechanism to identify and appropriately refer participants posing potential harm to themselves or others; however, the prevalence of reported clinician-diagnosed mental health disorders is similar to other studies of pediatric rheumatology patients. Second, generalizability of our results to the larger pediatric rheumatology patient population may be limited by several factors, including: the skewed demographic of the sample (predominately white, educated, and high-income); limitation of the study to JIA, JDM, and cSLE participants in order to leverage the PARTNERS member organizations which are focused on these diseases; and potential enrichment of patients with more disease burden (e.g. JDM and cSLE patients), comorbidities, and mental health issues than the general pediatric rheumatology population. Furthermore, while our varied recruitment approach including clinic advertisements, email listservs, and social media allowed us to reach a wider audience, it made it impossible to calculate response rate and gather information about those who did not complete the survey, also limiting assessment of generalizability of our results. Third, although we adjusted for patient characteristics in our comparisons between patient and parent responses, we could not compare pairs of patient respondents and parents from the same family due to the anonymous survey approach. Fourth, while our stratification of patients into adolescents and young adults is generally relevant for availability of pediatric vs adult medical and mental health services, we may not have captured differences in mental health experiences for the subgroup transitioning to adult care in their early 20s. Last, while we included the perspectives from parents of patients as young as 8 years old, the mental and behavioral health needs of these younger children were not directly captured in this survey. Despite these limitations, our study provides important and much needed insight into the mental health needs of youth with rheumatological disease, from their own perspective and from their parents.

## Conclusion

Youth with rheumatological disease in our study had high rates of reported mental health problems, both clinician- and self-diagnosed. Barriers to mental health treatment include disease-related and logistic factors, and mental health intervention strategies are needed to increase acceptance and accessibility of mental health intervention for these patients. Areas of focus identified by the experiences of patients and parents in this study include: 1) increased mental health education of patients, parents, medical and mental health providers; 2) optimization of partnerships for improved integration of medical and mental health care, and; 3) development and provision of disease-tailored mental health services and resources.

## Data Availability

Not applicable. Data used during the current study to support the study’s findings are included in the article as part of the review.

## Abbreviations

VAS: Visual Analog Score
JIA: Juvenile Idiopathic Arthritis
JDM: Juvenile Dermatomyositis
cSLE: childhood-onset Systemic Lupus Erythematous
CARRA: Childhood Arthritis & Rheumatology Research Alliance
CHOP: Children’s Hospital of Philadelphia
PARTNERS: Patients, Advocates, and Rheumatology Teams Network for Research and Service
PCORI: Patient-Centered Outcomes Research Institute
PR-COIN: Pediatric Rheumatology Care and Outcomes and Improvement Network
PCP: Primary Care Provider
