# Correction: Engaging patients and parents to improve mental health intervention for youth with rheumatological disease

**DOI:** 10.1186/s12969-024-00958-4

**Published:** 2024-02-16

**Authors:** Oluwatunmise A. Fawole, Michelle V. Reed, Julia G. Harris, Aimee Hersh, Martha Rodriguez, Karen Onel, Erica Lawson, Tamar Rubinstein, Kaveh Ardalan, Esi Morgan, Anne Paul, Judy Barlin, R. Paola Daly, Mitali Dave, Shannon Malloy, Shari Hume, Suzanne Schrandt, Laura Marrow, Angela Chapson, Donna Napoli, Michael Napoli, Miranda Moyer, Vincent Delgaizo, Ashley Danguecan, Emily von Scheven, Andrea Knight, for the CARRA Investigators

**Affiliations:** 1 Children’s Hospital of Philadelphia, Philadelphia, PA USA; 2 University of Pennsylvania, Philadelphia, PA USA; 3 New York University Grossman School of Medicine, New York, NY USA; 4 University of Missouri-Kansas City, Children’s Mercy Kansas City, Kansas City, MO USA; 5 University of Utah, Salt Lake City, UT USA; 6 Riley Hospital for Children at Indiana University Health, Indianapolis, IN USA; 7 Hospital for Special Surgery, Weill Cornell Medicine, New York, NY USA; 8 University of California San Francisco, San Francisco, CA USA; 9 Albert Einstein College of Medicine, Children’s Hospital at Montefiore, Bronx, NY USA; 10 Ann & Robert H. Lurie Children’s Hospital of Chicago, Chicago, IL USA; 11 Northwestern University Feinberg School of Medicine, Chicago, IL USA; 12 Duke University Medical Center, Durham, NC USA; 13 Cincinnati Children’s Hospital Medical Center, University of Cincinnati, Cincinnati, OH USA; 14 Lupus Foundation of America, Washington, D.C USA; 15 Cure JM Foundation, Leesburg, VA USA; 16 Arthritis Foundation, Atlanta, GA USA; 17 Patient-Centered Outcomes Research Institute, Washington, D.C USA; 18 The Childhood Arthritis and Rheumatology Research Alliance, Milwaukee, WI USA; 19 Division of Rheumatology, Hospital for Sick Children, Toronto, ON M5G1X8 Canada; 20 University of Toronto Faculty of Medicine, Toronto, ON Canada

Following publication of the original article [1], we have been notified that the third column heading in Tables 1, 2 and 3 was incorrect.

Initially published heading: Patient Report.

Corrected heading: Parent Report.

The original article has been corrected.
